# Enhancing Dental Alginate with *Syzygium aromaticum*, *Zingiber officinale* and Green Silver Nanoparticles: A Nature-Enhanced Approach for Superior Infection Control

**DOI:** 10.3390/gels10090600

**Published:** 2024-09-20

**Authors:** Lamia Singer, Leonie Beuter, Sabina Karacic, Gabriele Bierbaum, Jesenko Karacic, Christoph Bourauel

**Affiliations:** 1 Oral Technology, Dental School, Medical Faculty, University Hospital Bonn, 53111 Bonn, Germany; 2 Department of Orthodontics, Dental School, Medical Faculty, University Hospital Bonn, 53111 Bonn, Germany; 3 Institute of Medical Microbiology, Immunology and Parasitology, University Hospital Bonn, 53127 Bonn, Germany

**Keywords:** irreversible hydrocolloids, *Syzygium aromaticum*, *Zingiber officinale*, green synthesis, antimicrobial activity, nanotechnology

## Abstract

Objectives: The study addresses the challenge of cross-infection in dentistry, focusing on improving disinfection protocols for dental hydrocolloid gel materials. This research aimed to incorporate chlorhexidine, natural plant extracts, and green-synthesized silver nanoparticles (AgNPs) into dental alginate to enhance safety and hygiene standards in dental practices. Methods: Conventional dental alginate served as the control, with experimental groups including alginate modified with 0.2% chlorhexidine (CHX-group) and alginate incorporating AgNPs synthesized from *Syzygium aromaticum* (*SA*) and *Zingiber officinale* (*ZO*) extracts (SA + AgNPs and ZO + AgNPs groups). Plant extracts were analyzed via GC/MS to confirm their composition. UV–visible spectroscopy, SEM, and EDX characterized the synthesized AgNPs. Antimicrobial efficacy against *Candida albicans, Streptococcus mutans*, and methicillin-resistant and methicillin-sensitive *Staphylococcus aureus* was evaluated using agar well diffusion assays. The dimensional accuracy of alginate impressions was assessed according to ISO 21563:2021 standards. Results: Chemical analysis of *SA* and *ZO* extracts identified 60 and 43 active compounds, respectively, supporting their use in AgNP synthesis. UV–visible spectroscopy, SEM, and EDX confirmed the formation of spherical AgNPs using *SA* and *ZO* extracts. Modified groups showed inhibitory activity against *Streptococcus mutans* and methicillin-resistant (MRSA) and methicillin-sensitive *Staphylococcus aureus* (MSSA), in contrast to the unmodified control. Both AgNP-modified groups demonstrated efficacy comparable to the CHX-group against MRSA and MSSA, with SA + AgNP showing superior performance against *C. albicans*. The dimensional accuracy of all groups was within clinically acceptable ranges as reported in the literature (0.027–0.083 mm). Discussion: CHX, silver nitrate, and green-synthesized AgNPs present promising options for developing self-disinfecting alginate impression materials. Utilizing plant extracts in AgNP synthesis offers a safe, efficient, and synergistic approach between metal ions and phytotherapeutic agents. This approach could potentially enhance disinfection efficacy without compromising material performance, improving dental safety and hygiene.

## 1. Introduction

Dental alginate is a widely used impression material due to its affordability, ease of handling, and rapid setting time. While it provides less precise detail reproduction compared to elastomeric materials, it remains adequate for many clinical applications, including diagnostic models, impressions for opposing arches, provisional restorations, bleaching trays, and orthodontics [1]. Additionally, its hydrophilic nature makes alginate suitable for capturing impressions in moist environments, such as the oral cavity. However, alginate’s key limitation lies in its poor dimensional stability, which can lead to distortion over time, making it challenging to disinfect and store impressions without compromising accuracy [2]. This drawback has prompted research into modifications to improve alginate’s stability and longevity without sacrificing its favorable properties.

During the impression-taking process, all types of impression materials become contaminated with oral pathogens from the patient’s mouth. These microorganisms become embedded in the material during the procedure and remain on the surface of the impression once it sets [3,4]. Alginate impression material is especially prone to contamination because its hydrophilic nature and porous structure facilitate the penetration of microorganisms [5]. This vulnerability increases the risk of cross-contamination between clinical and laboratory personnel during handling [6].

To mitigate this problem, rinsing the impression under cold running water is recommended to remove visible debris such as saliva and, if present, the patient’s blood [1]. Although rinsing can significantly reduce surface contaminants, it is insufficient for complete decontamination. Studies have shown that many microorganisms can persist after rinsing, which underscores the necessity of a thorough disinfection process to ensure the impression is safe for further use [7]. Additionally, inadequate disinfection can compromise the accuracy of the impression and lead to potential health risks, making the development of more effective and stable disinfectant methods a key focus in dental material research [1].

Various methods are used to disinfect alginate impressions, including the spray technique or immersion in a disinfectant solution. Both spraying and immersion techniques have drawbacks when disinfecting alginate impression material [8,9]. While immersion ensures thorough disinfection of the entire impression, there is a risk of the disinfectant solution being absorbed by the hydrophilic alginate material, causing it to expand. This expansion can lead to dimensional changes and compromise the mechanical properties of the impression material [10]. On the other hand, the spraying technique might not effectively reach all areas, particularly undercuts, potentially leaving some parts insufficiently disinfected. Additionally, both methods primarily disinfect the surface, which can result in microorganisms becoming trapped within the material, posing a potential risk for incomplete disinfection [4].

A novel approach has emerged recently, which involves integrating the disinfectant into the material beforehand, either by blending it with the alginate powder or the mixing liquid. This intrinsic disinfection approach could eliminate the necessity for an extra step following impression-making and save time. Moreover, it ensures the disinfection process is not overlooked [6] and guarantees the disinfection of the entire material rather than just the surface [5,11]. Previous studies have demonstrated that adding chlorhexidine, the gold standard of antimicrobial agents in dentistry, to alginates does not alter the accuracy of impressions [12]. Furthermore, the addition of silver nanoparticles does not negatively affect the physical properties of the alginate impression material, such as the detail reproduction [13].

Silver nanoparticles (AgNPs) have gained attention over the years for their remarkable antimicrobial activity [14]. Their small size, large surface area, and unique physicochemical properties make them effective against a broad spectrum of bacteria, viruses, and fungi [14]. Green synthesis of nanoparticles using medicinal plants has emerged as an eco-friendly and sustainable method to produce AgNPs [14]. Plant extracts contain various phytochemicals acting as reducing and capping agents, facilitating the reduction of silver ions to nanoparticles. These extracts not only serve as reducing agents but also contribute additional antimicrobial compounds, enhancing the antimicrobial potency of the synthesized nanoparticles [15,16]. This approach employs the bioactive potential of plants while promoting a greener and cost-effective means to create antimicrobial agents with broad applications in healthcare and numerous other fields [15,16].

*Syzygium aromaticum* (cloves) and *Zingiber officinale* (ginger) are well-known medicinal plants with a rich history of use in traditional medicine. Cloves have been employed in traditional Chinese and Ayurvedic medicine for centuries, and they are recognized for their antimicrobial and antiseptic properties, primarily due to the compound eugenol [17,18]. This compound also has analgesic effects, making cloves beneficial in pain relief. Ginger, similarly, has been valued for its therapeutic benefits over centuries, with its rhizome used to address various health conditions [19]. It contains a complex array of volatile and non-volatile compounds, including gingerol, a phenolic compound that is crucial for its antioxidative and antimicrobial activities [20]. These properties of ginger make it effective in reducing inflammation, combating oxidative stress, and aiding digestion.

Based on the aforementioned information, this study aimed to introduce an innovative approach to developing self-disinfecting dental alginate by incorporating silver nanoparticles synthesized from plant extracts. Our previous research, which was the first to use *Boswellia sacra* extract for this purpose, demonstrated effective antimicrobial properties while preserving the clinical performance of alginate [21]. Building on these findings, this study aimed to explore and compare the effectiveness of silver nanoparticles synthesized from clove (*Syzygium aromaticum*) and ginger (*Zingiber officinale*) extracts. By using these two plant extracts, we evaluated their synergistic actions and how they differ based on their chemical constituents. Nanoparticle formation was confirmed through visual color changes, UV–Vis spectroscopy, scanning electron microscopy (SEM), and energy-dispersive X-ray spectroscopy. The antimicrobial efficacy and dimensional changes of alginate modified with these extracts were compared against unmodified alginate (negative control) and chlorhexidine-modified alginate (positive control).

## 2. Results and Discussion

### 2.1. Results

#### 2.1.1. Chemical Analysis

Gas chromatography/mass spectrometry (GC/MS) revealed the presence of 60 volatile and semi-volatile active compounds for the *S. aromaticum* extract and 43 volatile and semi-volatile compounds for *Z. officinale* (Table 1 and Table 2).

#### 2.1.2. Characterization of the Ag Nanoparticles

##### Color Change and UV–Visible Spectroscopy Analysis

**Figure 1:**
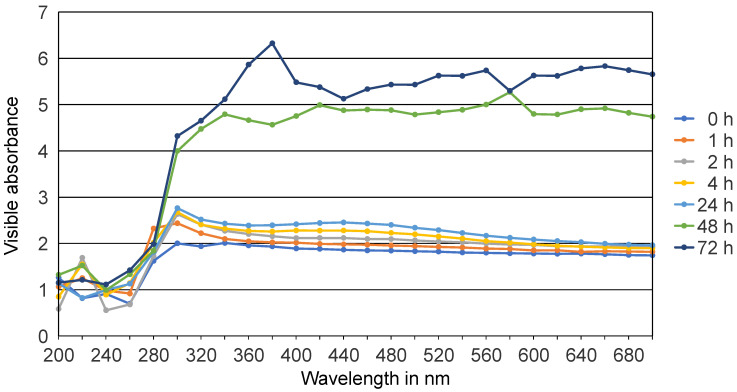
UV–Vis absorption spectra of Ag NPs green synthesized by *S. aromaticum* extract at different time intervals.

**Figure 2:**
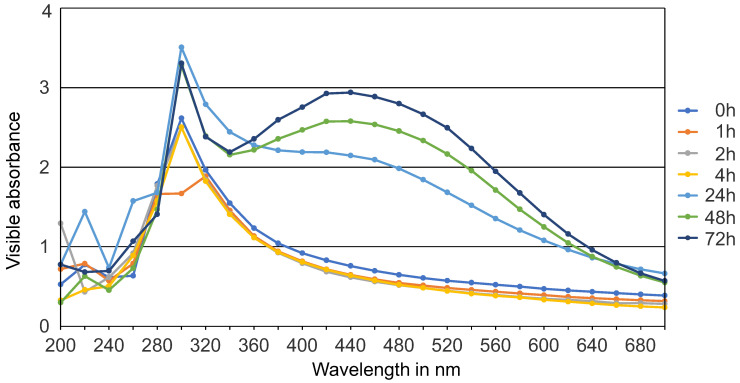
UV–Vis absorption spectra of Ag NPs green synthesized by *Z. officinale* extract at different time intervals.

The biological synthesis of AgNPs using *Syzygium aromaticum* extract and *Zingiber officinale* extract as a reducing and stabilizing agent was confirmed primarily by the color change from white to light brown and then dark brown. Silver nanoparticle surface plasmon excitation causes the color change in the solution, which is the key and notable evidence for the formation of Ag NPs. Several UV–Vis absorption spectra were measured after 0, 1, 2, 4, 24, 48 h, and 3 days intervals (Figure 1 and Figure 2). For both solutions, a gradual increase in the intensity as a function of time was observed. The maximum intensity was observed after 3 days. An absorption peak between 300 nm and 320 nm was detected, likely associated with the *Syzygium aromaticum* and *Zingiber officinale* extracts. Another absorption peak, ranging from 400 nm to 440 nm, corresponds to the localized surface plasmon resonance of the AgNPs [21]. Localized surface plasmon resonance (LSPR) is a phenomenon where conduction electrons on the surface of metallic nanoparticles, like silver or gold, collectively oscillate in resonance with incident light. This oscillation amplifies the electromagnetic field at the nanoparticle surface, resulting in strong light absorption and scattering at specific wavelengths [22]. Moreover, *Syzygium aromaticum* (clove) showed another peak at 560 nm, which could indicate the presence of silver aggregates or larger particles.

##### Scanning Electron Microscopy—EDX

**Figure 3:**
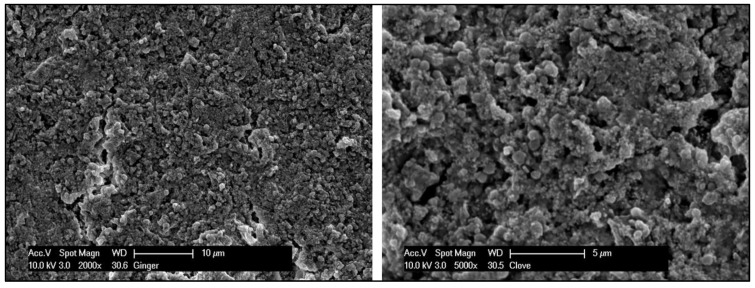
SEM magnified images (2000× and 5000×) confirming the formation of spherical nanoparticles and nanoclusters using *SA* + AgNPs.

**Figure 4:**
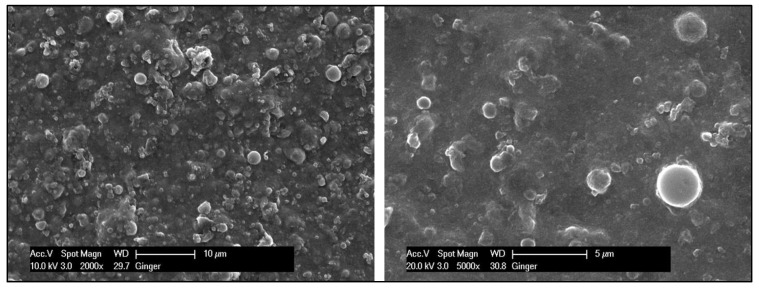
SEM magnified images (2000× and 5000×) confirming the formation of spherical nanoparticles and nanoclusters using *ZO* + AgNPs.

**Figure 5:**
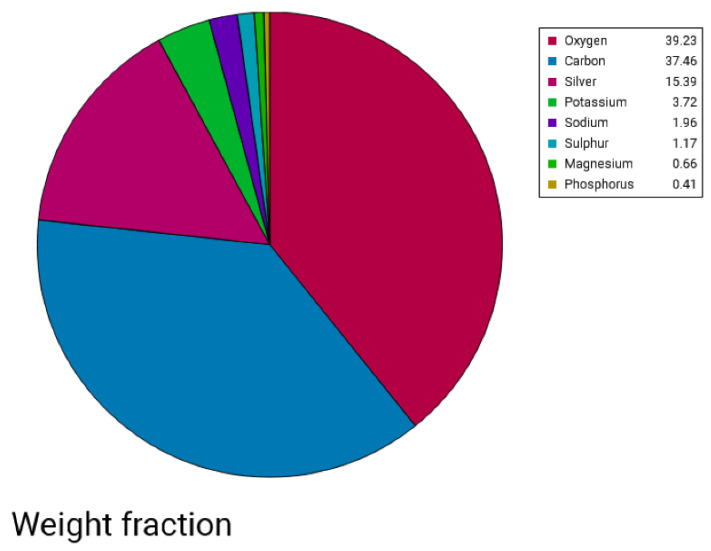
Pie chart showing the elements identified in the *SA* + AgNPs mixture using EDX.

**Figure 6:**
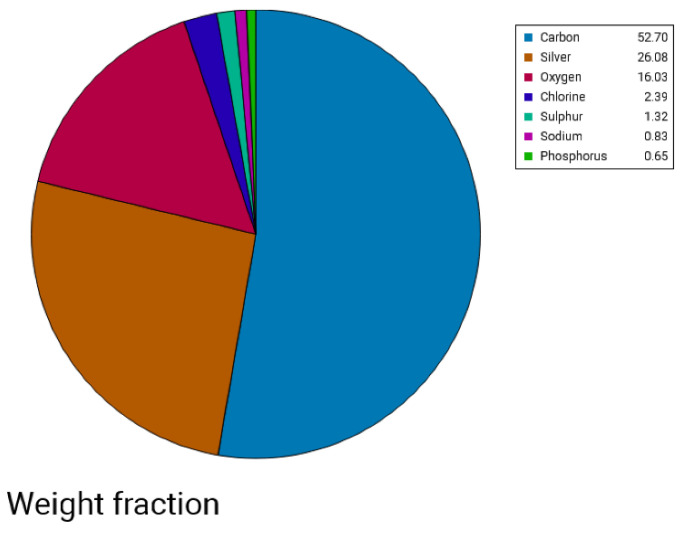
Pie chart showing the elements identified in the *ZO + AgNPs mixture* using EDX.

The morphological images of Ag NPs produced by green synthesis are shown in Figure 3 and Figure 4. The results indicate the presence of spherical nanoparticles, as well as clusters of spherical nanoparticles with different diameters ranging from 900 to 400 nm and up to 1 µm for the Ginger extract, while much smaller nanoparticles were obtained using clove extract (50–100 nm), and more aggregations were observed (Figure 3 and Figure 4). EDX confirmed the generation of silver nanoparticles and clusters along with the presence of potassium sodium, sulfur, magnesium, phosphorus, oxygen, and chlorine (Figure 5 and Figure 6).

#### 2.1.3. Antimicrobial Activity

**Figure 7:**
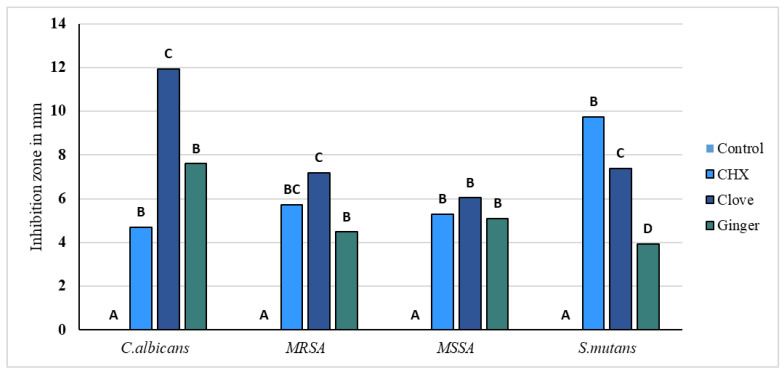
Bar chart representing the mean inhibition zone values of the four tested alginate groups against *S. mutans*, *MRSA*, *MSSA*, *and C. albicans.* Groups that do not share same letter are significantly different.

Agar diffusion tests were performed in triplicates. The variables showed a parametric distribution for *S. mutans*, MRSA, and MSSA, and thus one way ANOVA was used to test the antimicrobial effect of *SA +* AgNPs, CHX, *ZO* + AgNPs, and control groups against these different microbial strains followed by Tukey’s post hoc test for pairwise comparison between the tested groups (Figure 7). The ANOVA indicated that there was a statistically significant antibacterial effect of the three modified groups of alginates against all strains, *p*-value < 0.001. Post hoc comparison using Tukey’s test indicated that the mean values of the three modified groups showed significant antibacterial activity compared to the unmodified control.

For *Candida albicans*, *SA* + AgNPs (clove) exhibited the highest mean inhibitory effect (11.92 mm ± 2. 3), significantly outperforming CHX (4.71 mm ± 1.5) and *ZO* + AgNPs (ginger) (7.60 mm ± 0.3), with the control group showing no inhibition effect at all. In the case of MRSA, clove and CHX demonstrated the highest mean inhibition zones (7.18 mm ± 1.2 and 5.74 mm ± 0.8, respectively). Moreover, *ZO* + AgNPs (ginger) (4.48 mm ± 0.5) and CHX were comparable and significantly higher than the control. For *MSSA*, *SA* + AgNPs (clove) and ginger and CHX had comparable inhibitory effects to each other (6.04 mm ± 1.9, 5.08 mm ± 0.4, and 5.30 mm ± 0.7, respectively) while significantly higher than the control. For *S. mutans*, CHX showed significantly the highest mean inhibition zones (9.75 mm ± 2), followed by *SA* + AgNPs (clove) (7.38 mm ± 1.3) and then *ZO* + AgNPs (ginger) (3.93 mm ± 0.4). Overall, *SA* + AgNPs (clove) generally provided the most substantial inhibition across most pathogens, whereas CHX showed robust effects, especially for *S. mutans*. *ZO* + AgNPs (ginger), while effective, was generally less potent than *SA* + AgNPs (clove) and CHX.

#### 2.1.4. Dimensional Accuracy

**Figure 8:**
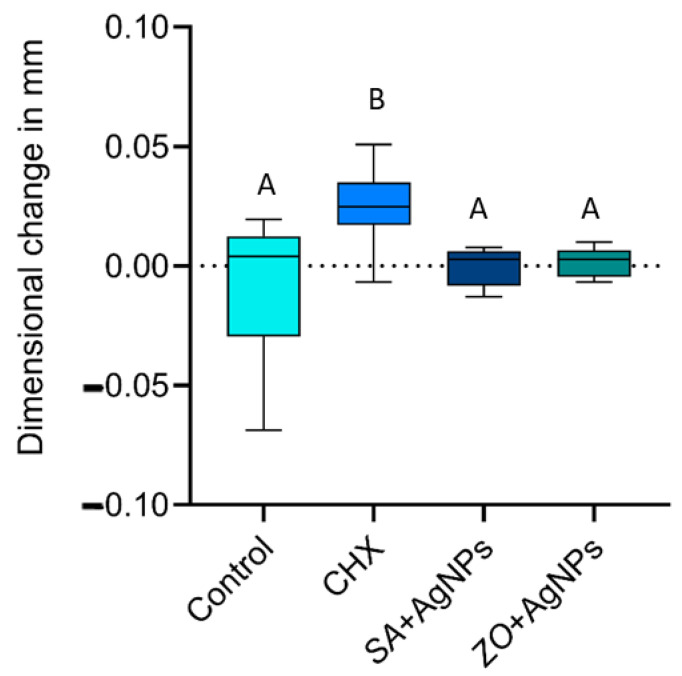
Interval plot illustrating the mean total dimensional changes in millimeters in the vertical and horizontal dimensions and 95% confidence interval of all tested groups. Groups that do not share same letter are significantly different.

Data showed parametric distribution, and thus, the mean and standard deviation values of the dimensional changes in the tested materials are represented in Figure 8. The results indicated that the mean dimensional changes in mm were 0.007 ± 0.028 mm (negative control), 0.025 ± 0.016 mm (CHX), 0.000 ± 0.008 mm (*SA* + AgNPs), and 0.002 ± 0.006 mm (*ZO* + AgNPs). From the results, it is clear that there was a statistically significant difference between dimensional changes in the four materials (*p*-value < 0.001). CHX showed the statistically significantly highest mean dimensional changes. The specimens were significantly larger than the other tested groups but still within the acceptable range found in the literature.

### 2.2. Discussion

Utilizing natural plant extracts for modifying dental materials against microorganisms is important because of their inherent properties and eco-friendly nature [23]. Unlike chemicals, natural plant extracts often possess antimicrobial properties, making them effective against microorganisms. They also minimize potential harm to human health and the environment by using non-harmful solvents and reducing energy consumption. Furthermore, these methods often result in nanoparticles with enhanced biocompatibility and stability, making them well-suited for biomedical applications in dentistry [24].

In the present study, the confirmation of nanoparticle synthesis was achieved through UV–Vis spectrophotometry and visual observation, where the initially light-colored solution underwent a discernible change to a brown hue. UV–visible spectroscopy stands as a straightforward and extensively utilized analytical method for monitoring the generation of AgNPs. When subjected to an electromagnetic field, the conducting electrons found in the outermost orbital of metal NPs collectively oscillate in resonance with specific wavelengths, resulting in the manifestation of surface plasmon resonance (SPR). The excitation of SPR is accountable for the development of color and absorbance in a colloidal solution of AgNPs [22].

Typically, the confirmation of silver nitrate reduction into AgNPs is validated by observing SPR peaks in the visible range from 380 to 450 nm [25]. Small silver nanospheres normally absorb light most strongly around 380–400 nm, while larger spheres, approximately 100 nm in diameter, have a broader absorption peak that shifts towards longer wavelengths, approaching 500 nm. In clove samples, an additional peak suggests aggregation of silver nanoparticles. Aggregation causes the LSPR peak of silver nanoparticles to shift to longer wavelengths, typically between 500 and 700 nm, influenced by aggregation degree and environmental factors. This shift results from plasmon interactions between closely spaced particles, altering their optical properties. Larger spheres may also exhibit a secondary peak at shorter wavelengths due to quadrupole resonance alongside dipole resonance [26].

Subsequent SEM analysis revealed the existence of synthesized nanoparticles, presenting as both spherical and oval in shape. Predominantly, these nanoparticles exhibited aggregation, although a few individual microparticles and nanoparticles were also identified. The Energy Dispersive X-ray Spectroscopy (EDX) results for a drop of a mixture of silver nitrate added to clove plant extract and another drop of silver nitrate added to ginger plant extract indicated the presence of silver, carbon, oxygen, magnesium, phosphorus, sodium, potassium, and chlorine. The detection of silver confirms the successful reduction of silver ions to silver nanoparticles by the phytochemicals present in the clove and ginger extracts, which act as reducing and stabilizing agents [27,28].

GC-MS (gas chromatography–mass spectrometry) analysis was employed to identify and analyze the compounds in plant extracts responsible for the reduction of metal ions to nanoparticles. In the case of clove extract (*Syzygium aromaticum*), GC-MS identified eugenol as a major phenolic compound that played a crucial role in reducing silver ions to silver nanoparticles. Additionally, other compounds, such as flavonoids, tannins, and terpenoids, were found to aid in the stabilization of the nanoparticles [29]. For ginger extract (*Zingiber officinale*), GC-MS revealed gingerol and other bioactive compounds that contributed to the reduction in metal ions and stabilization of nanoparticles [30].

The agar diffusion assays revealed an antimicrobial activity of CHX and both AgNP groups against *C. albicans*, *S. mutans*, MRSA, and MSSA. CHX showed significantly better antimicrobial action against *S. mutans* in comparison to the other three groups. *SA* + AgNPs and *ZO* + AgNPs were significantly more active against all tested strains compared to the unmodified control group. There was a significant difference between *SA* + AgNPs and *ZO* + AgNPs against *C. albians*, *S. mutans,* and MRSA, in which the modification with *SA* + AgNPs (clove extract) had the stronger action. Clove was the only modification that significantly surpassed CHX (the gold standard of antimicrobials) against *C. albicans*. The strong antifungal activity of clove extract is also shown in a study by Jardón-Romero et al., who found a growth-inhibitory effect of clove extract on *C. albicans* [18].

A synergistic action between the phytochemicals of the plant extracts and silver ions may be an enhancing factor for the antimicrobial action. Gas chromatography and mass spectrometry identified several phytochemical constituents, which are part of one of the three big families of secondary plant metabolites (terpenoids, flavones, and alkaloids).

The most detected chemical constituents in the *S. aromaticum* extract belong to the terpenoids (e.g., α-Cedrol, β-Caryophyllen, and Carveol), but polyphenols and flavonoids were also detected. Several mechanisms of action are attributed to them, like the destruction of the cell membrane, which leads to an increase in permeability [31]. Carveol, for example, affects the membrane integrity of *S. aureus* cells and leads to the loss of potassium ions [32]. The main component of clove extract is the phenylpropanoid eugenol, which is mainly responsible for its pharmacological action [18]. Eugenol also leads to depolarization and permeabilization of the cell membrane of staphylococci [33], and its presence inside the extract may be one reason for the enhanced antimicrobial action in comparison to the *ZO* + AgNPs group. Flavonoids (e.g., 3,5,7-trimethoxy-flavone, Swertisin, and 2′,4′-Dimethoxy-3-hydroxy-6-methylflavone) are the most detected chemical group of the *Zingiber officinale* extract besides terpenoids and other polyphenols. Flavonoids lead to bacterial cell death by disruption of the cell membrane and leakage of cellular content. The DNA and energy metabolisms are also affected [31].

Utilizing plant extracts in the green synthesis of nanoparticles yields smaller particles, offering improved dispersion and a heightened surface-to-volume ratio, which have been shown to enhance antimicrobial activity [34]. The size, shape, and structure of nanoparticles dictate their reactivity and durability [35]. Although the precise mechanisms driving the action of silver nanoparticles are not fully elucidated [36], existing research indicates several influential pathways. Silver nanoparticles are able to pass through cell walls and disrupt cytoplasmic membranes [14,37]. Moreover, these nanoparticles interact with proteins, causing the breakdown of disulfide bridges and subsequent protein aggregation [14,38].

Crucially, the antimicrobial efficacy of silver nanoparticles is linked to the release of positively charged silver ions. These ions engage electrostatically with negatively charged cell walls, entering cells and prompting the generation of reactive oxygen species, instigating oxidative stress. This oxidative stress inhibits DNA and mRNA synthesis, protein biosynthesis, and cell wall formation. These cumulative effects impede cell division, leading ultimately to cell death [39].

Significantly, the effectiveness of silver nanoparticles varies concerning Gram-negative and Gram-positive bacteria. Gram-negative bacteria, possessing a thinner peptidoglycan layer, exhibit higher susceptibility to silver nanoparticle actions due to their easily penetrable cell walls. In contrast, Gram-positive bacteria, with a thicker peptidoglycan layer forming a more robust structure, are comparatively harder to penetrate [40].

Besides its antimicrobial action, silver nitrate is also toxic to humans in a dose-dependent manner. Oral ingestion of more than 2 g can be fatal due to the reaction with chloride, which results in the formation of insoluble silver chloride and leads to electrolyte imbalance [41]. The concentration of silver nitrate used for the green synthesis in this study was very low (0.2%). One alginate impression, made with the plant extract–silver nanoparticles mix, contains a maximum of 0.0566 g silver nitrate, which equals 2.83% of the toxic dose. Additionally, the liquid used to mix the alginate no longer contains pure silver nitrate because it undergoes a reaction with the plant extract to form nanoparticles, so the concentration is even lower.

To achieve precise gypsum casts, the dimensional accuracy of an impression material is crucial. Impression materials must reproduce accurate measurements and remain dimensionally stable over time, although this accuracy can be influenced by water evaporation and syneresis. The stability of alginate gels depends on storage conditions and their constituents, while water loss is affected by factors such as entropy, osmotic pressure, Gibbs free energy, diffusion kinetics, and environmental gradients [42,43]. ISO 21563:2021 [44] does not specify requirements for alginate impression materials. However, the range between 0.027 and 0.083 mm marginal discrepancy is clinically acceptable for cast and ceramic restorations [43,45].

All the tested groups demonstrated values within acceptable ranges. The two groups modified with green silver nanoparticles were not significantly different from the control group or each other. However, CHX showed significantly different results compared to all groups, indicating slight expansion. These findings suggest that CHX may affect the dissolution of the calcium sulfate reactor, which releases calcium ions crucial for forming the insoluble calcium alginate gel. Lower concentrations of calcium ions were observed to enhance the swelling capacity of alginate beads, resulting in increased material expansion [46,47].

This study has several limitations that should be considered. The chemical compositions of the plant extracts used here may be modulated by the growth conditions and origins of the plants. Additionally, the ratio of silver nitrate to plant extract was fixed at 5:1, but other ratios might produce nanoparticles of different sizes and efficacy. The dimensional changes in the alginate impressions were measured using a metallic mold without simulating oral conditions, such as the presence of saliva, which may impact the performance of the material in real clinical applications. These limitations suggest that further studies with a broader range of extracts, different ratios, and testing conditions more closely related to clinical situations are necessary to better understand the potential of these materials in dental applications.

## 3. Conclusions

This study offers a new approach to addressing cross-infection from dental impressions in dentistry through the integration of chlorhexidine, natural plant extracts, and green-synthesized silver nanoparticles into dental alginate materials. After conducting thorough analyses of plant extract compositions, confirming the formation of effective silver nanoparticles, and assessing antimicrobial performance against key pathogens, significant advancements were observed. The modified alginate formulations exhibited superior antimicrobial efficacy against *S. mutans*, MRSA, MSSA, and *C. albicans* compared to conventional unmodified materials while maintaining the dimensional accuracy required for clinical use. These findings highlight the potential of using natural antimicrobial agents and eco-friendly nanoparticle synthesis techniques to enhance infection control measures in dental settings.

## 4. Materials and Methods

### 4.1. Materials

For the study, a dust-free, normal-setting dental alginate (Blueprint Xcreme^®^, Dentsply DeTrey GmbH, Konstanz, Germany) was used as the base material. Antimicrobial modifications were achieved by preparing solutions of chlorhexidine digluconate (Sigma-Aldrich, St. Louis, MO, USA) and ≥99% silver nitrate powder (Sigma-Aldrich, St. Louis, MO, USA) dissolved in distilled water. Additionally, plant extracts were prepared from commercially available *Syzygium aromaticum* (clove buds) and *Zingiber officinale* (ginger rhizome).

### 4.2. Preparation of Antimicrobial Modification Solutions

Firstly, the chlorhexidine solution for the modification of alginate was prepared by mixing 1 g of chlorhexidine digluconate solution with 500 mL of distilled water to achieve a concentration of 0.2%. For the preparation of the silver nitrate solution needed for the green synthesis of silver nanoparticles, 1 g of silver nitrate powder was dissolved in 500 mL of distilled water. The 0.2% silver nitrate solution was subsequently combined with aqueous extracts derived from *Syzygium aromaticum* (clove) or *Zingiber officinale* (ginger) [21].

**Figure 9:**
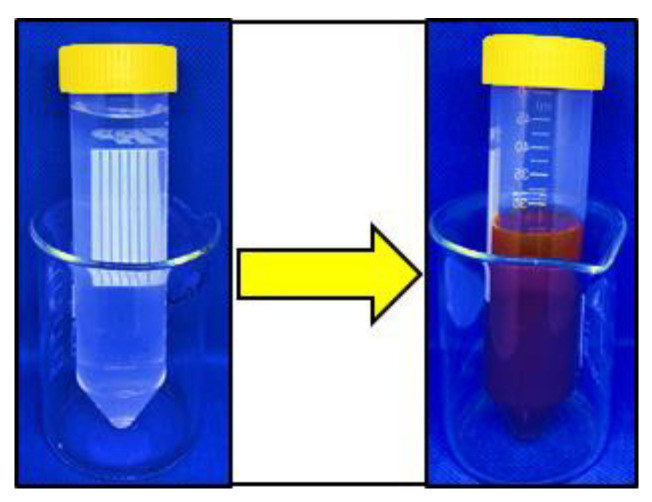
Color change in the *Syzygium aromaticum* extract + AgNO_3_ mixture, transitioning from lighter to darker hues, indicating the reduction and formation of silver nanoparticles.

To obtain the aqueous extract of *S. aromaticum*, 25 g of pulverized clove buds were soaked in 150 mL of a solvent mixture consisting of 80% distilled water and 20% ethanol for 72 h. The mixture was then filtered through Whatman^®^ filter paper Nr. 1 to separate the liquid extract from the solid residue. Similarly, an extract of *Zingiber officinale* (ginger) was prepared using the same method: 25 g of ground ginger rhizomes were soaked in 150 mL of the same solvent mixture for 72 h and filtered through the same type of filter paper. This process ensures the extraction of soluble compounds from both plant materials. The resulting aqueous extracts were subsequently used for the eco-friendly synthesis of two different silver nanoparticles. Two mixtures were prepared, each composed of 25 mL of a 0.2% silver nitrate solution and 5 mL of an aqueous plant extract (in a 5:1 ratio), and incubated for 36 h. The color change from a lighter to a darker hue signaled the successful formation of silver nanoparticles (Figure 9). Subsequently, the mixtures of silver nitrate solution and plant extracts were employed to modify two additional groups of alginate [48].

### 4.3. Experimental Groups

The experiments included the following groups:

1. Negative Control: Alginate was mixed with distilled water.
2. Positive Control: Alginate was mixed with a 0.2% chlorhexidine solution instead of water.
3. Modified Groups:
  ○ In one group, alginate was mixed with a solution of silver nitrate and the prepared clove extract (5:1).
  ○ In the other group, alginate was mixed with a solution of silver nitrate and the prepared ginger extract (5:1).

For each group, the alginate was manually mixed until a homogeneous mass was achieved, adhering to the manufacturer’s instructions regarding the powder-to-liquid ratio.

### 4.4. Chemical Analysis of Plant Extracts

At the Agricultural Research Center in Giza, Egypt, 1 mL of each clove extract and ginger extract was analyzed for their chemical constituents using gas chromatography and mass spectrometry (GC/MS). The gas chromatograph comprises a polar Agilent HP 5% phenylmethylpolysiloxane and a capillary column with an inner diameter of 0.25 mm and a thickness of 0.25 µm. The detector temperature was maintained at 25 °C, while the injection temperature was set to 200 °C. Helium served as the carrier gas, with a linear velocity of 1 mL/min. The mass spectrometer was operated within a range of 50–800 m/z, and the interface temperature was adjusted to 250 °C. The identification of compounds involved comparing their relative retention times with those listed in the NIST and WILEY databases. Additionally, the spectral data were cross-referenced with the literature data [21,49].

### 4.5. Characterization of Silver Nanoparticles

#### 4.5.1. Scanning Electron Microscopy–Energy-Dispersive X-ray Spectroscopy

A scanning electron microscope (SEM, Philips XL 30, Philips, Eindhoven, The Netherlands) was utilized to examine the structure, shape, and size of the formed silver nanoparticles. A drop of the clove–silver particle mixture and the ginger–silver nanoparticle mixture was applied as a thin film on aluminum stubs with conductive tape, coated with carbon, and allowed to dry. The samples were examined under vacuum at a voltage of 25 kV and a spot size of 3. The synthesized silver nanoparticles were photographed at 5000× magnification and measured using Image J software (Wayne Rasband (NIH), Version 1.53k). On the other hand, the elemental composition of the sample was analyzed using energy-dispersive X-ray (EDX) spectroscopy attached to the SEM (Genesis 4000, EDAX AMETEK GmbH, Taunusstein, Germany). EDX samples were examined at a magnification of 2000×, and the machine operated at 25 kV [50].

#### 4.5.2. UV–Vis Spectroscopy

UV–Vis spectroscopy (UviLine 9400, Schott, Mainz, Germany) was used to examine the color change in plant extract–silver solutions. Aliquots of 1 mL from both solutions were measured at different time intervals. Immediately after combining the plant extract–silver solution, the absorption spectrum was measured. Subsequent measurements were taken at 1, 2, 4, 24, 48, and 72 h. Distilled water served as a reference. The scanning speed for the samples in the range of 200 to 700 nm was 475 nm/min. The optical path was 1 cm at room temperature [15,51].

### 4.6. Analysis of Antimicrobial Activity

The antimicrobial activity was analyzed using the agar well diffusion test following EUCAST guidelines. The test organisms included *Streptococcus mutans* (DSMZ 20523)*,* methicillin-resistant *Staphylococcus aureus* (MRSA, USA 300 NRS384), methicillin-sensitive *Staphylococcus aureus* (MSSA), *Staphylococcus aureus* (SG511), and *Candida albicans* (DSM 70014). Strains were cultured from glycerol stock in liquid Mueller–Hinton (MH) medium for 24 h at 37 °C, while *S. mutans* was incubated at 37 °C with 5% CO_2_. The overnight culture of each strain was measured with a UV–Vis spectrometer at 600 nm, and optical density was adjusted to 0.1.

MH plates (Mueller–Hinton agar plates, VWR) were flooded with 1 mL of the culture solution and incubated for 10 min. Following incubation, any remaining culture was removed by pipetting. Four 8 mm diameter wells were then punched into each agar plate, and the freshly mixed alginate samples were filled into the wells using sterile disposable plastic spatulas. The agar plates were incubated at 37 ± 1 °C for 24 h for general growth conditions, while Streptococcus mutans were incubated at 37 °C with 5% CO_2_ to mimic its natural, CO_2_-rich environment and promote optimal growth. This specific condition ensures an accurate evaluation of its antimicrobial response. All experiments were performed in triplicate.

**Figure 10:**
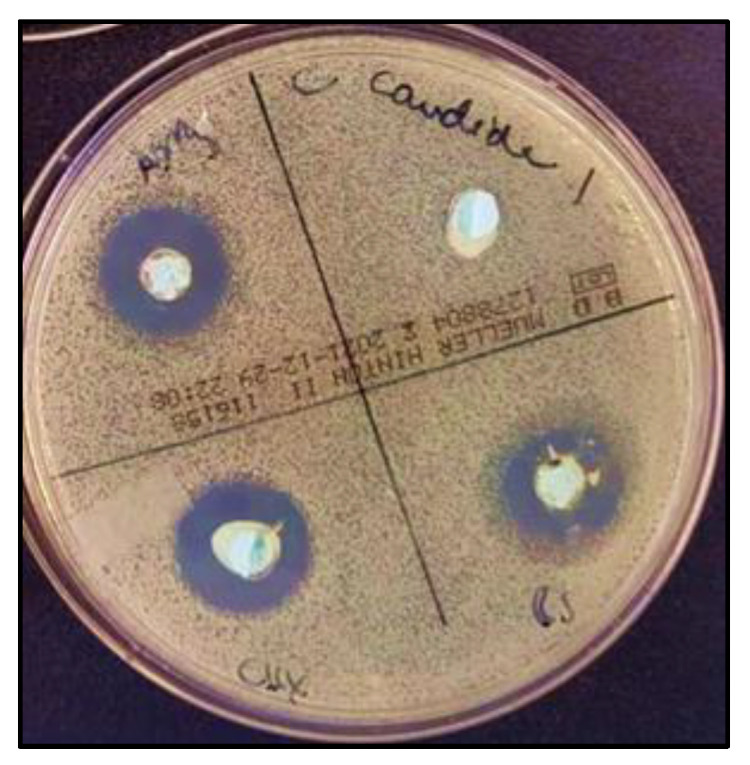
Inhibition zones observed on agar plates for the four tested groups against *Candida albicans*.

Subsequently, the plates were photographed using a tripod and a support structure with reference points to ensure standardization. The digital images taken were utilized to measure the inhibition zones around the alginate samples using Image J software (Wayne Rasband (NIH), Version 1.53k). Each inhibition zone was measured in four directions, and the average values were recorded after subtraction of the diameter of the wells, Figure 10 [21].

### 4.7. Dimensional Changes

The test followed ADA specification No. 18 [52] and ISO 21563:2021 [44], with minor modifications to the metallic mold for measuring both the ‘X’ and ‘Y’ axes of the specimens. The mold featured vertical and horizontal lines, creating three squares that intersect to produce reference points for measuring the vertical and horizontal dimensions at the top and bottom of the sample. Each group (*n* = 10) was mixed according to the manufacturer’s instructions, filled into the mold, covered with a metal plate, and weighted to simulate the force applied during impression making. After setting, specimens were photographed at 18× magnification using a stereomicroscope. For each specimen, four measurements were taken along the X and Y axes between the reference points at the top and bottom of the sample, with each dimension measured three times to calculate the mean value [48]. This process was also applied to the mold without impressions for comparison. Dimensional changes were calculated using the ISO 4823:2000 [53] formula:

ΔL = (L1 − L1/L1) × 100(1)

where L1 is the measured distance on the mold, and L2 is the measured distance on the samples.

### 4.8. Statistical Analysis

The results are expressed as mean values together with their corresponding standard deviations (SD). The Shapiro–Wilk normality test was utilized to assess the normal distribution of variables. All quantitative variables exhibited a parametric distribution; consequently, a one-way analysis of variance (ANOVA) was applied to compare among the groups. When the ANOVA test yielded significance, Tukey’s post hoc test was employed for pairwise comparisons between the groups. The threshold for statistical significance was set at *p* ≤ 0.05. The statistical analyses were conducted using GraphPad Prism 10.1.2.

## Figures and Tables

**Table 1:** Results of GC/MS analysis of *S. aromaticum* extract.

NO.	Retention Time	Name	Area Sum %
1	5.322	α-Cedrol	1.75
2	5.683	Caryophyllene	0.42
3	5.741	Methyleugenol	0.33
4	6.446	Gallic acid	1.89
5	6.725	Patchoulol	1.18
6	7.053	2-Heptanol	1.16
7	7.701	Longiborneol	0,47
8	7.807	Isobornyl acetate	0.77
9	8.012	Caffeic acid	0.96
11	8.316	2-Hexadecanol	1.28
12	8.632	Salicylic acid	0.34
10	8.8	Phytol	0.7
13	9.14	Carveol	2.2
14	9.513	β-Curcumene	0.89
15	9.792	Chavico	1.14
16	9.86	Cinnamyl alcohol	1.45
17	10.46	Flavone,3′,4′,6,7-tetra methoxy	0.71
18	10.57	p-Cymen-7-ol	1.03
19	10.74	Cubebol	0.77
20	10.85	Ylangene	0.47
21	11.12	Eugenol	29.46
22	11.25	γ-Muurolene	1.61
23	11.28	α-copaen	0.8
24	11.61	Isoeugenol	0.74
25	11.79	β-Caryophyllen	10.12
26	12.08	α-Humulene	2.26
27	12.13	β-Elemen	1.08
28	12.27	γ-Cadinene	0.58
29	12.51	Alloaromadendren	0.67
30	12.74	δ-Cadinene	3.72
31	12.86	α-Cubebene	0.42
32	12.90	α-Cedrene	0.39
33	12.95	Retinol	0.38
34	13.11	Caryophyllene epoxide	0.58
35	13.16	α-Ionol	0,92
36	13.37	Widdrol	0.77
37	13.38	Elemol	1
38	13.41	Ledene	0.77
39	13.53	β-Patchoulene	2.57
40	13.88	α-Ylangene	0.59
41	13.94	Sclareol	0.39
42	13.99	β-Ionone	0.43
43	14.12	Epiglobulol	0.3
44	14.17	Thunbergol	0.67
45	14,31	Spathulenol	1.11
46	14.43	4′-Hydroxy-5-methoxy flavone	1.17
47	15.61	Shyobunol	0.87
48	15.86	Syringic acid	0.92
49	16.24	β Carotene	1.87
50	16.64	Afromosin 7-O-glucoside	0.86
51	16.89	Flavone, 5-hydroxy-3,3′,4′,6,7-pentamethoxy-	0.52
52	18.09	3′,4′,5′,5,6,7-Hexamethoxyflavone	1.15
53	18.31	Oleyl oleate	2.05
54	18.63	Ascaridole	1.06
55	19.36	Vitexin	1.54
56	20.74	Homovanillic Acid	1.52
57	21.10	Flavone, 3,5,7-tri methoxy-	1.18
58	21.61	γ-Selinene	1.12
59	22.77	Dehydrodieugenol	0.74
60	23.08	Geranyl isovalerate	1.18

**Table 2:** Results of GC/MS analysis of *Z. officinale* extract.

NO.	Retention Time	Name	Area Sum %
1	3.908	7-Hydroxy-4-methyl-3-phenyl coumarin	2.23
2	4.486	2′,5′-Dimethoxyflavone	1.18
3	4.662	2-Hydroxychalcone	1.26
4	4.851	3′,4′,5′,5,6,7-Hexamethoxyflavone	1.21
5	5.089	Clovane	1.32
6	5.458	Luteolin 6-C-glucoside	0.51
7	5.782	Swertisin	1.14
8	6.389	7,3′,4′,5′-Tetramethoxyflavanone	1.54
9	6.651	3,2′,4′,5′-Tetramethoxyflavone	0.72
10	6.987	Isopulegol	1.53
11	7.463	Cubebol.	0.75
12	7.643	7,2′,4′-Trimethoxyflavone	0.64
13	8.041	Gallic acid	1.44
14	8.316	Gentisic acid	1.23
15	8.685	α-Cedrol	0.63
16	8.947	Epicubebol	2.25
17	9.157	Caryophyllene epoxide	1.17
18	9.509	Sclareo	1.46
19	9.636	3,6,3′,4′-Tetramethoxyflavone	0.31
20	10.428	Casticin	1.01
21	11.174	Patchoulane	1.15
22	12.027	Caryophyllene oxide	5.21
23	12.294	3,7,8,2′-Tetramethoxyflavone	2.16
24	13.254	2-Cyclohexen-1-one	1.38
25	13.356	2,6-Dimethylphenol	2.03
26	13.717	2′,4′-Dimethoxy-3-hydroxy-6-methyl flavone	1.85
27	13.975	Calderol	1.49
28	14.5	3,6,2′,3′-Tetramethoxyflavone	1.34
29	15.009	Vanillic acid	2.51
30	15.173	Flavone, 3,5,7-trimethoxy-	1.88
31	15.456	Gardenin	0.9
32	15.841	Flavone, 4′,5,6,7-tetramethoxy	3.2
33	16.522	5,7,2′-Trimethoxyflavone	2.57
34	16.653	Oleic Acid	3.78
35	16.961	Farnesol	2.08
36	17.219	Corymbolone	1.84
37	17.724	Digoxigenin	4.04
38	18.282	Isolongifolol	1.93
39	18.54	Decanoic acid	2.19
40	19.274	β Carotene	0.75
41	21.571	β-Ionone	10.19
42	22.543	β-Stigmasterol	16.75
43	23.064	Betulin	5.25

## Data Availability

The data presented in this study are openly available in the article.
